# Ring D-Modified and Highly Reduced Angucyclinones From Marine Sediment-Derived *Streptomyces* sp.

**DOI:** 10.3389/fchem.2021.756962

**Published:** 2021-10-12

**Authors:** Lin Guo, Qiaoli Yang, Guangfei Wang, Shumin Zhang, Ming Liu, Xiaohong Pan, Gennaro Pescitelli, Zeping Xie

**Affiliations:** ^1^ School of Pharmacy, Binzhou Medical University, Yantai, China; ^2^ College of Life Sciences, Yantai University, Yantai, China; ^3^ Dipartimento di Chimica e Chimica Industriale, Università di Pisa, Pisa, Italy

**Keywords:** angucyclinone, nonaromatic D-ring, tetraphene, atropisomerism, antibiotic activity, TDDFT calculations, absolute configuration

## Abstract

Angucyclines and angucyclinones represent the largest family of type II PKS-engineered natural products. Chemical analysis of a marine *Streptomyces* sp. KCB-132 yielded three new members, actetrophenone A (1) and actetrophenols A–B (2–3). Their structures were elucidated by NMR spectroscopy, X-ray crystallography and CD calculations. Actetrophenone A (1) is the first representative of a novel-type angucyclinone bearing a nonaromatic D-ring. Actetrophenol A (2) features a highly reduced and aromatized four-ring system, which is unprecedented for natural products. While (*R*_*a*_)- and (*S*_*a*_)-actetrophenol B (3) bear an unprecedented *N*-acetyltryptamine-substituted tetraphene core skeleton, this is the first report of a pair of atropisomeric isomers in the angucyclinone family. Actetrophenol A (2) exhibits remarkable antibiotic activity, notably including potent activity to multiple resistant *Staphylococcus aureus* and *Enterococcus faecium* with MIC values of 4 μg/ml, in contrast, the positive control antimicrobial agent penicillin was inactive up to 32 μg/ml.

## Introduction

Angucyclines and angucyclinones, characterized by an unsymmetrically assembled benz[*a*]anthraquinone frame, represent by far the largest family of type II PKS-engineered natural products (Kharel et al., 2012). This family of antibiotics has attracted much attention due to their broad biological activities and remarkable structural diversity that is mainly derived from oxidation, hydroxylation and glycosylation at various positions (Bringmann et al., 2005; Shaaban et al., 2012; Xie et al., 2016), and additionally, rearrangement of A-, B- and C-ring (Ma et al., 2015; Liu et al., 2019; Wu et al., 2019). However, an aromatic D-ring and a high number of oxygen functions on the tetracyclic backbone remain the common features of their structures, and neither ring D-modified nor highly reduced angucyclin(on)es have been reported as yet (Cabrera-Afonso et al., 2018).

**FIGURE 1:**
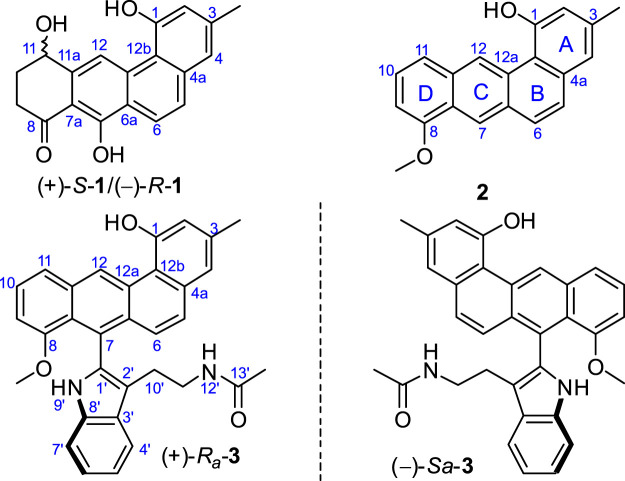
Chemical structures of compounds 1‒3.

As part of our research on the discovery of new-type angucyclinones from marine sediment-derived bacteria, we reported several angucyclinones featuring C-ring cleavage and expansion, produced by *Streptomyces pratensis* KCB-132 (Zhang et al., 2017; Zhang et al., 2019). Recently, a comprehensive LC-MS-based analysis of this strain identified a group of metabolites possessing previously unreported UV chromophores. The subsequent purification of the fraction containing these components led to the discovery of three further unique angucyclinone-derived polyketides, with ring D-modified (1) and highly reduced (2 and 3) structural characteristics (Figure 1). We describe herein the isolation and structural elucidation of these new metabolites, named actetrophenone A (1) and actetrophenols A–B (2–3). In addition, the atropisomerism of 3 is discussed.

## Results and Discussion

Actetrophenone A (1) was isolated as a light brown powder. Its molecular formula was predicted to be C_19_H_16_O_4_ with twelve degrees of unsaturation, based on high-resolution electrospray ionization (HR-ESI) mass spectrometry ([M + H]^+^ at *m/z* 309.1122, calculated 309.1127). The ^1^H NMR spectrum of 1 in DMSO-*d*_*6*_ showed a methyl singlet at *δ*_H_ 2.43, an oxygenated methine signal at *δ*_H_ 4.90, two pairs of geminal protons at *δ*_H_ 2.92, 2.78 and at *δ*_H_ 2.27, 2.09, as well as five aromatic protons at *δ*_H_ 9.38, 8.11, 7.68, 7.26 and 7.01 (Table 1). The ^13^C NMR spectrum revealed the expected 19 carbon signals, classified as a methyl, an oxymethine (*δ*_C_ 67.3), two methylenes, five aromatic methines (*δ*_C_ 115.2–127.1), nine aromatic tertiary carbons (*δ*_C_ 110.7–157.5), and a carbonyl carbon (*δ*_C_ 205.2) with the aid of a DEPT-135 measurement, accounting for eight degrees of unsaturation, which required four rings in the scaffold of 1.

**TABLE 1:** ^1^H and^13^C NMR data for 1-3 in DMSO-*d*_*6*_ ^a^.

Position	1		2		3	
	*δ*_H_ mult (*J*, Hz)	*δ*_C_ *^b^*	*δ*_H_ mult (*J*, Hz)	*δ*_C_ *^b^*	*δ*_H_ mult (*J*, Hz)	*δ*_C_ *^b^*
1	—	157.5, C	—	156.6, C	—	154.6, C
2	7.01, d (1.7)	115.2, CH	7.07, s	115.2, CH	6.90, s	115.9, CH
3	—	139.1, C	—	137.0, C	—	137.9, C
4	7.26, s	120.2, CH	7.22, s	119.9, CH	7.20, s	121.8, CH
4a	—	136.6, C	—	134.3, C	—	134.5, C
5	7.68, d (9.1)	127.1, CH	7.56, d (9.0)	127.3, CH	7.37, d (9.4)	128.4, CH
6	8.11, d (9.1)	120.8, CH	7.84, d (9.0)	128.0, CH	7.41, d (9.4)	125.4, CH
6a	—	120.8, C	—	130.1, C	—	131.6, C
7	—	159.8, C	8.64, s	120.1, CH	—	128.7, C
7a	—	110.7, C	—	122.9, C	—	123.4, C
8	—	205.2, C	—	154.6, C	—	155.5, C
9	2.92, ddd (17.8, 7.3, 5.1)	35.2, CH_2_	6.97, d (7.9)	103.0, CH	6.87, d (7.9)	106.2, CH
	2.78, ddd (17.8, 8.9, 5.1)					
10	2.27, m	32.1, CH_2_	7.47, t (7.9)	125.6, CH	7.46, t (7.9)	125.2, CH
	2.09, m					
11	4.90, m	67.3, CH	7.68, d (7.9)	120.9, CH	7.82, d (7.9)	123.4, CH
11a	—	142.8, C	—	132.5, C	—	133.6, C
12	9.38, s	117.3, CH	10.16, s	126.5, CH	10.24, s	129.8, CH
12a	—	136.6, C	—	129.0, C	—	123.4, C
12b	—	116.9, C	—	115.6, C	—	116.2, C
1-OH	10.72, s	—	—	—	—	—
3-CH_3_	2.43, s	—	2.44, s	20.9, CH_3_	2.49, s	21.2, CH_3_
7-OH	13.72, s	—	—	—	—	—
8-OCH_3_	—	—	4.05, s	55.5, CH_3_	3.54, s	56.5, CH_3_
11-OH	5.60, d (5.1)	—	—	—	—	—
1’	—	—	—	—	—	136.0, C
2’	—	—	—	—	—	119.5, C
3’	—	—	—	—	—	128.1, C
4’	—	—	—	—	7.72, d (7.8)	118.5, CH
5’	—	—	—	—	7.23, t (7.8)	119.5, CH
6’	—	—	—	—	7.27, t (7.8)	121.6, CH
7’	—	—	—	—	7.43, d (7.8)	110.6, CH
8’	—	—	—	—	—	136.0, C
10’	—	—	—	—	2.90, ddd (14.6, 6.1, 4.3)	24.5, CH_2_
					2.50, ddd (14.6, 8.9, 5.0)	
11’	—	—	—	—	3.64, m	39.3, CH_2_
					3.30, ddd (17.4, 8.9, 4.3)	
13’	—	—	—	—	—	169.7, C
9’-NH	—	—	—	—	8.16, s	—
12’-NH	—	—	—	—	5.72, s	—
13’-CH_3_	—	—	—	—	1.37, s	22.7, CH_3_

^a^ 600 MHz for ^1^H NMR and 150 MHz for ^13^C NMR.

b Numbers of attached protons were determined by analysis of 2D spectra.

**FIGURE 2:**
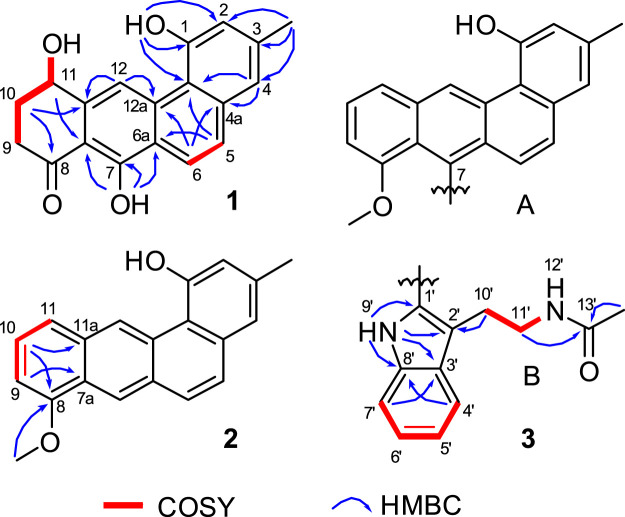
Selected HMBC and ^1^H−^1^H COSY correlations of 1, D-ring in 2, and *N*-acetyltryptamine motif in 3.

Inspection of ^1^H-^1^H COSY NMR data led to identification of two isolated spin systems (Figure 2). The first spin system showed correlations from H_2_-9 to 11-OH, which was extended to include two tertiary carbons C-7a/C-11a and a carbonyl carbon C-8 based on HMBC correlations from H_2_-10 to C-8/C-11a and from H-11 to C-7a to construct a 4-hydroxy-2-cyclohexen-1-one ring. The second spin system containing two aromatic protons, H-5 and H-6, showed HMBC couplings from H-5 to C-6a/C-12b and from H-6 to C-4a/C-12a to form a benzene unit and was further expanded by HMBC correlations from 1-OH to C-1/C-2/C-12b, from 3-CH_3_ to C-2/C-3/C-4 and from H-4 to C-4a/C-12b, revealing the presence of a 3-methylnaphthalen-1-ol ring. Additional HMBC signals with key correlations from 7-OH to C-6a/C-7/C-7a and from H-12 to C-11a/C-12a fused the two ring systems together and resulted in the planar structure of 1.

**FIGURE 3:**
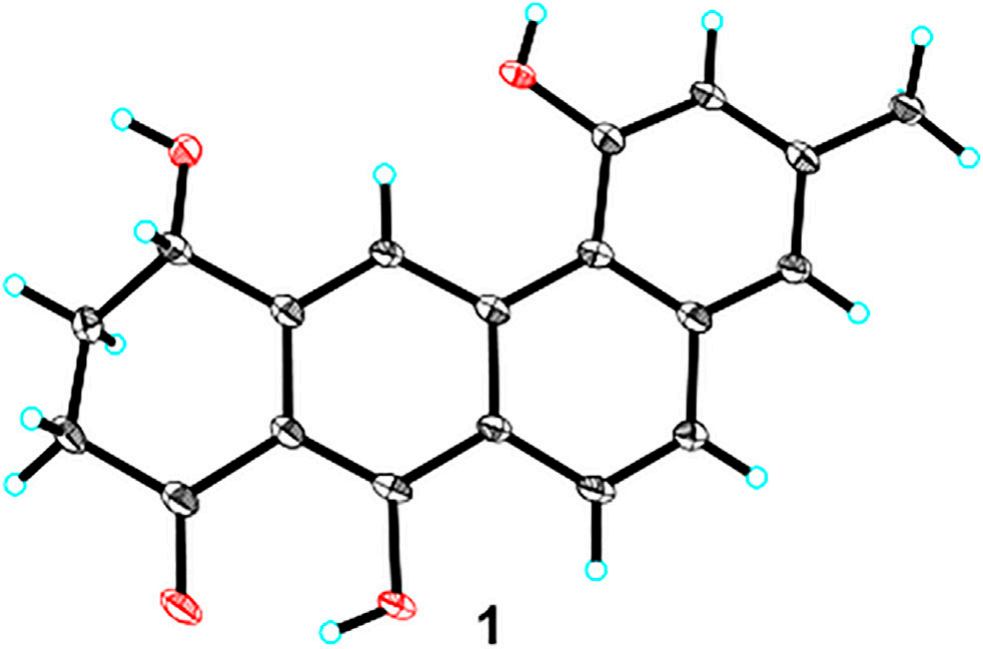
X-ray crystal structure of 1.

Since ring D-modified angucycline was unprecedented in natural products, and since ring C-aromatized representative was very rare in the angucycline family, crystals of 1 were subjected to X-ray crystallographic analysis (CCDC 2045687). Slow evaporation of a concentrated solution containing 1 in a MeOH/CH_2_Cl_2_ mixture furnished X-ray quality crystals. The X-ray crystal data corroborated the structure assignment of 1 and provided a centrosymmetric space group *P*-1, supporting a racemic mixture (Figure 3). Interestingly enough, the crystal structure of 1 belongs to the relatively rare case of “solid solution” with both enantiomers appearing in the asymmetric unit with disordered chiral centre (Rekis, 2020).

Actetrophenol A (2) was purified as a light brown powder. Its molecular formula was assigned as C_20_H_16_O_2_ based on HR-ESI mass spectrometry ([M + H]^+^ at *m/z* 289.1200, calculated 289.1228). The NMR data of 2 (Table 1) were quite similar to those of 1 and showed main changes in ring D (Figure 2). In 2, the nonaromatic D-ring previously seen in 1 was replaced with a conventional 1,2,3-trisubstituted benzene ring, as evidenced by COSY cross-peaks between H-10 and H-9/H-11 alongside HMBC contacts from H-9 to C-7a, from H-10 to C-8/C-11a and from 8-OCH_3_ to C-8. Furthermore, an additional change in the NMR data of 2 was observed in C-7, whose chemical shift was significantly downfield-shifted from *δ*_C_ 159.8 to 120.1, which indicated the lack of an oxygen atom in line with the molecular formula, and thus completed the planar structure of 2.

Actetrophenol B (3) was also obtained as a light brown powder. Its molecular formula was determined to be C_32_H_28_N_2_O_3_ based on HR-ESI mass spectrometry ([M + Na]^+^ at *m/z* 511.1991, calculated 511.1998). Careful comparison of the ^1^H and ^13^C NMR spectra of 2 and 3 showed superimposable resonances for the tetraphene part as subunit A (Figure 2; Table 1). Further analysis of 2D NMR data of 3 delineated the presence of a *N*-acetyltryptamine motif as subunit B. In particular, COSY NMR data quickly established the H-4’/H-5’/H-6’/H-7’ spin system. Combined HMBC correlations from H-4’ to C-8’, from H-7’ to C-3’ and from 9’-NH to C-1’/C-2’/C-3’/C-8’, allowed the construction of an indole ring. A further COSY sequence of signals from H-10’ to 12’-NH along with HMBC correlations from H-10’ to C-2’ and from both H-11’ and 13’-CH_3_ to the amide carbonyl carbon C-13’ (*δ*_C_ 169.7) permitted the attachment of an *N*-ethylacetamide side chain in C-2’ of the indole ring.

**FIGURE 4:**
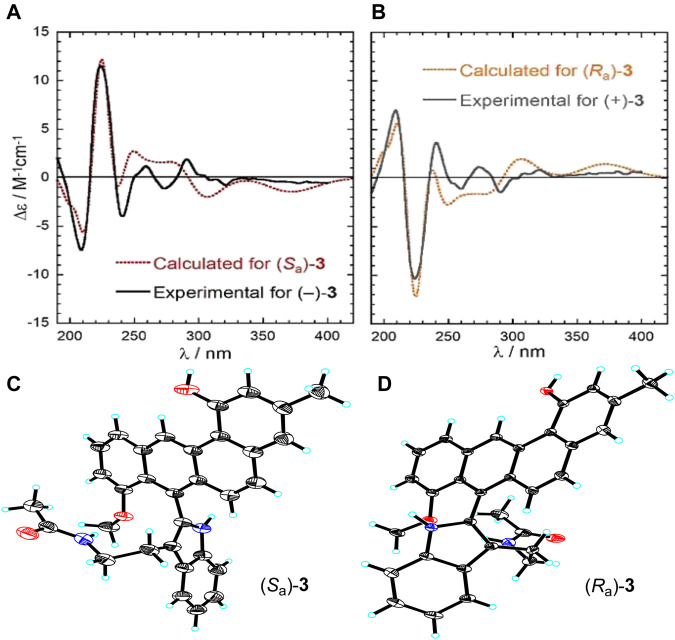
**(A)** Experimental and calculated ECD spectra of (−)-(*S*_a_)-3, **(B)** experimental and calculated ECD spectra of (+)-(*R*_a_)-3, **(C)** X-ray crystal structure of (*S*_a_)-3, **(D)** X-ray crystal structure of (*R*_a_)-3.

**FIGURE 5:**
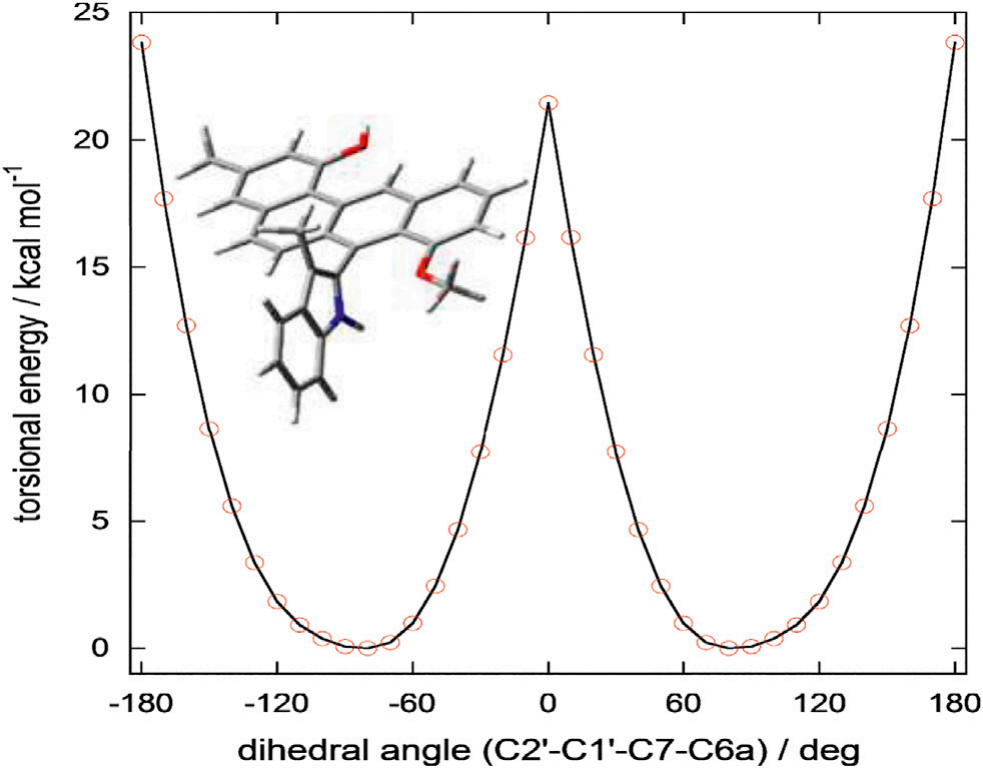
Torsional energy scan of a simplified model of 3 run at ωB97X-D/6-31+G(d) level in vacuo. In the truncated model trunc-3, the substituent at C2’ in 3 (CH_2_CH_2_NHCOCH_3_) was replaced by a methyl group (see Figure). The substituent CH_2_CH_2_NHCOCH_3_ is capable of stabilizing the energy minima by intramolecular hydrogen bonding (see Supplementary Figure S1); on the contrary, it hardly affects the energy of the transition state because it folds away from the benz[*a*]anthracene ring. Therefore, the energy barrier of trunc-3 offers a lower estimate of the racemization barrier in 3. The plot was obtained by running two independent torsional energy scans varying the C2’-C1’-C7-C6a dihedral angle in steps of 10° and considering for each value of the dihedral angle the lowest energy value.

At this point of the structure elucidation two aromatic tertiary carbons, C-7 in subunit A and C-1’ in subunit B, were still loose ends. Considering the molecular formula of **3**, subunits A and B had to be linked between C-1’ and C-7, unequivocally, although no obvious correlation was observed between 9’-NH and C-7 in the HMBC spectrum as it might have been expected. However, this assignment was supported by X-ray diffraction experiment (CCDC 2045689). The X-ray analysis confirmed the structure assigned for 3 but also indicated its racemic nature from a centrosymmetric space group *P*121/*c*1 (Figures 4C,D). Consequently, resolution of single atropisomers was achieved by HPLC equipped with a chiral column (CHIRALCEL OD-H). The interconversion (racemization) barrier between the two atropisomers was estimated by DFT calculations at ωB97X-D/6-31+G(d) level and amounted to ≈ 20 kcal mol^−1^ (Figure 5).

Finally, the absolute configurations of the two enantiomers were determined by electronic circular dichroism (ECD) calculations run with TDDFT method at CAM-B3LYP/def2-TZVP//ωB97X-D/6-311+G(d,p)/PCM level (see Supporting Information for details) (Pescitelli and Bruhn, 2016; Superchi et al., 2018). The ECD spectra of these two isomers recorded in acetonitrile exhibited a perfect mirror-image relationship and were in good agreement with the TDDFT calculated spectra of the possible atropisomers with *S*_a_ and *R*_a_ configurations, respectively (Figures 4A,B).

The three isolated compounds and their angucyclinone analog 8-*O*-methyltetrangulol were evaluated for activity against Gram-positive (*Enterococcus faecalis* ATCC 29212, *Staphylococcus aureus* CMCC 26002, *Bacillus subtilis* CMCC 63501, *Bacillus cereus* CMCC 32210, *Salmonella* CMCC 50094) and Gram-negative (*Escherichia coli* CMCC 44102) bacteria, and “ESKAPE” pathogens (*Staphylococcus aureus*, *Enterococcus faecium*, *Pseudomonas aeruginosa*, *Acinetobacter baumannii*). Notably, actetrophenol A (2) showed moderate to good antimicrobial activity against Gram-positive strains with MIC values ranging from <1 to 16 μg/ml, and also exhibited potent activities against multiple resistant *Staphylococcus aureus* and *Enterococcus faecium* with MIC values of 4 μg/ml, while the positive control antimicrobial agent penicillin was inactive in the tested concentration range (MIC > 32 μg/ml) (Table 2). In contrast, compounds 1, 3 and 8-*O*-methyltetrangulol exhibited no activity towards all tested strains up to 32 μg/ml. Furthermore, compounds 1 and 2 demonstrated equivalent activities against THP-1 cells with IC_50_ values of 10.07 and 12.35 *μ*M, respectively (Table 3).

**TABLE 2:** *In vitro* antibacterial activity (MIC in μg/ml) of compounds 1–3 and 8-*O*-methyltetrangulol.

Organism	MIC(*μ*g/mL)					
	1	2	(*R*_*a*_)-3	(*S*_*a*_)-3	8-*O*-methyltetrangulol	penicillin
*Enterococcus faecalis*ATCC 29212	>32	16	>32	>32	>32	4
*Staphylococcus aureus* CMCC 26002	>32	<1	>32	>32	>32	<1
*Bacillus subtilis* CMCC 63501	>32	4	>32	>32	>32	<1
*Bacillus cereus* CMCC 32210	>32	2	>32	>32	>32	>32
*Salmonella*CMCC 50094	>32	16	>32	>32	>32	8
*Escherichia coli*CMCC 44102	>32	>32	>32	>32	>32	>32
multiple resistant*Staphylococcus aureus* *^a^*	>32	4	>32	>32	>32	>32
multiple resistant*Enterococcus faecium* *^b^*	>32	4	>32	>32	>32	>32
multiple resistant*Pseudomonas aeruginosa*	>32	>32	>32	>32	>32	>32
multiple resistant*Acinetobacter baumannii*	>32	>32	>32	>32	>32	>32

a Resistance to *β*-lactam [penicillin (MIC ≥ 0.5 μg/ml), oxacillin (MIC ≥ 4 μg/ml)], aminoglycoside [gentamycin (MIC ≥ 16 μg/ml)], 4-quinolone [ciprofloxacin(MIC ≥ 8 μg/ml), levofloxacin (MIC ≥ 8 μg/ml), moxifloxacin(MIC = 4 μg/ml)], macrolide [erythromycin(MIC ≥ 8 μg/ml)], lincosamide [clindamycin(MIC ≥ 8 μg/ml)], tetracycline (MIC ≥ 16 μg/ml) and rifampicin (MIC ≥ 32 *μ*g/ml)antibiotics.

b Resistance to *β*-lactam [penicillin (MIC ≥ 64 μg/ml), ampicillin (MIC ≥ 32 μg/ml)], 4-quinolone [ciprofloxacin (MIC ≥ 8 μg/ml), levofloxacin (MIC ≥ 8 μg/ml)], macrolide [erythromycin (MIC ≥ 8 μg/ml)], and vancomycin(MIC ≥ 32 μg/ml) antibiotics.

**TABLE 3:** Cytotoxicities of compounds 1–3.

Cell line	IC_50_ (μM)			
	1	2	(*R*_*a*_)-3	(*R*_*s*_)-3
THP-1	10.07	12.35	>60	>60

## Experimental Section

### General Experimental Procedures

Opitical rotations were measured on an Autopol VI (Serial #91058) manufactured by Rudolph Research Analytical, Hackettstown, NJ, United States CD spectra were recorded using a JASCO J-810 spectropolarimeter. NMR spectra were measured by a Bruker AVANCE IIITM 600 spectrometer. ESI-HRMS were recorded on a Waters ACQUITY UPLC I Class-Vion IMS QTof spectrometer. Single crystal X-ray crystallography was determined on SMART APEX II DUO X-ray single crystal diffractometer using Cu K*α* radiation. Preparative HPLC was performed on a Waters 2,489 series instrument with a UV/Visible detector, using a reversed-phase C18 column (Phenomenex, 250 mm × 21.2 mm, 5 μm). Chiral HPLC was carried out on an Agilent 1,260 liquid chromatograph, utilized chiral analytical columns (CHIRALCEL OD-H column, 4.6 mm × 250 mm, 5 μm).

### Cultivation and Culture Extraction

*Streptomyces* sp. strain KCB-132 was isolated from a sediment sample collected off Kiaochow Bay, China, as described previously. The sequence is deposited in GenBank under accession no. KX033803. The strain KCB-132 was cultured in seawater-based ISP2 medium (5 g of malt extract, 4 g of yeast extract, 4 g of glucose, 500 ml of deionized water and 500 ml of seawater, pH 7.8) with the addition of 50 μM lanthanum chloride, at a total volume of 21.6 L (72 × 0.3 L), for 10 days at 28 °C. The culture broth was filtered to provide filtrate and mycelium. The filtrate was absorbed onto XAD-16 amberlite resin, and the resin was eluted with methanol, then dry out methanol under reduced pressure, the resulting aqueous layer was extracted with ethyl acetate, while the mycelium was extracted by ethyl acetate under ultrasonic radiation directly, both ethyl acetate phases were combined to yield 7.2 g crude extract.

### Isolation of Actetrophenone A (1) and Actetrophenols A–B (2-3)

The extract (7.2 g) was fractioned by silica gel column chromatography (CC, 40 g) and eluted with a step gradient of CH_2_Cl_2_ and MeOH. The CH_2_Cl_2_/MeOH 95:5 fraction (4.7 g) was subjected to ODS CC with a stepwise gradient of MeOH-H_2_O (10:90→100:0) to provide ten fractions (Fr.C1-Fr.C10), and Fr.C10 (15 mg) was purified by preparative HPLC (Gemini, C18, 21.2 mm × 250 mm, 5 μm, UV = 210 nm), eluting with 80% MeOH in H_2_O to afford actetrophenol A (Two, 6.0 mg, *t*_R_ = 37.7 min). Fr.C8 (140 mg) was purified by the same preparative HPLC system eluting with 70% MeOH in H_2_O to give actetrophenone A (One, 1.7 mg, *t*_R_ = 34.0 min) and actetrophenol B (Three, 5.6 mg, *t*_R_ = 49.0 min). Chiral resolution of 3 was performed on Agilent analytical HPLC system (CHIRALCEL OD-H column, 4.6 mm × 250 mm, 5 μm, iso-Pro-OH/n-Hexane = 20:80, 1.0 ml/min, UV = 210 nm) to obtain optically pure (*R*_a_)Three (1.6 mg, *t*_R_ = 21.0 min) and (*S*_a_)-3 (1.7 mg, *t*_R_ = 33.0 min).

Actetrophenone A (1): light brown powder; UV (Acetonitrile) *λ*_max_ (log *ε*) 200 (2.5), 219 (2.6), 274 (2.9), 298 (2.5), 324 (2.6), 343 (2.3) nm; 1D and 2D-NMR (600 MHz, DMSO-*d*_*6*_), see Table 1; HRESIMS [M + H]^+^ at *m/z* 309.1122 (calculated 309.1127).

Actetrophenol A (2): light brown powder; UV (Acetonitrile) *λ*_max_ (log *ε*) 230 (3.5), 258 (3.2), 273 (3.3), 283 (3.3), 301 (3.0), 313 (3.2) nm; 1D and 2D-NMR (600 MHz, DMSO-*d*_*6*_), see Table 1; HRESIMS [M + H]^+^ at *m/z* 289.1200 (calculated 289.1228).

(*R*_a_)-Actetrophenol B (*R*_a_ **-**3): light brown powder; [*α*]_D_^25^ + 34.4 (Acetonitrile, *c* 0.05); UV (Acetonitrile) *λ*_max_ (log *ε*) 230 (3.5), 260 (3.2), 277 (3.3), 286 (3.3), 306 (3.0), 318 (3.2) nm; 1D and 2D-NMR (600 MHz, CDCl_3_), see Table 1; HRESIMS [M + Na]^+^, *m/z* 511.1991 (calculated 511.1998).

(*S*_a_)-Actetrophenol B (*S*_a_-3): light brown powder; [*α*]_D_^25^ − 28.8 (Acetonitrile, *c* 0.05); UV (Acetonitrile) *λ*_max_ (log *ε*) 230 (3.5), 260 (3.2), 277 (3.3), 286 (3.3), 306 (3.0), 318 (3.2) nm; 1D and 2D-NMR (600 MHz, CDCl_3_), see Table 1; HRESIMS [M + Na]^+^, *m/z* 511.1991 (calculated 511.1998).

### Computational Section

MMFF and DFT calculations were run with Spartan’18 (Wavefunction, Inc., Irvine CA, 2019), with standard parameters and convergence criteria. TDDFT calculations were run with Gaussian’16 (Rev. C.01, Gaussian, Inc., Wallingford CT, 2013), with default grids and convergence criteria. Conformational searches were run on Three with the Monte Carlo algorithm implemented in Spartan’18 using Merck molecular force field (MMFF). All structures thus obtained were first optimized with DFT method using B97X-D functional and 6-31G(d) basis set in vacuo, and then re-optimized using B97X-D functional and 6-311+G(d,p) basis set including SMD solvent model for acetonitrile. Torsional energy scans were run on a simplified model of Three with the substituent at C-2’ replaced by a methyl group, by varying the dihedral angle relative to the biaryl axis by 10 deg steps; calculations were run at B97X-D/6-31+G(d) level in vacuo. TDDFT calculations were run using various functionals (B3LYP, CAM-B3LYP, M06-2X) and def2-TZVP basis set, including 32 excited states and the IEF-PCM solvent model for acetonitrile. CAM-B3LYP and M06-2X functionals led to consistent results, while B3LYP results were in poorer agreement with the experiment. ECD spectra were generated using the program SpecDis (v. 1.71, Bruhn T., Schaumlöffel A., Hemberger Y., Pescitelli G., Berlin, Germany, 2017, https://specdis-software.jimdo.com/), by applying a Gaussian band shape with 0.26 eV exponential half-width, from dipole-length rotational strengths. The calculated spectra were red shifted by 18 nm and scaled by a factor 10 for better comparison with the experimental spectra. Boltzmann populations were estimated at 300K from internal energies calculated at B97X-D/6-311+G(d,p)/SMD level. The conformers with Boltzmann population >2% at 300K were included in the calculations, which amounted to 4 conformers for 3. The structures and relative energies are shown in Supplementary Figure S1. All stable conformers had an intramolecular hydrogen bond between the amide N-H and 8-OMe and a similar value of the aryl-aryl dihedral angle C2’-C1’-C7-C6a ≈ 71–74°.

### Antimicrobial Bioassay

The antimicrobial assays of compounds 1–3 and 8-*O*-methyltetrangulol were tested against Gram-positive (*Enterococcus faecalis* ATCC 29212, *Staphylococcus aureus* CMCC 26002, *Bacillus subtilis* CMCC 63501, *Bacillus cereus* CMCC 32210, and *Salmonella* CMCC 50094) and Gram-negative (*Escherichia coli* CMCC 44102) bacteria, and resistant strains (*Staphylococcus aureus*, *Enterococcus faecium*, *Pseudomonas aeruginosa*, *Acinetobacter baumannii*) using a microplate assay. Penicillin was used as positive controls against fungi and bacteria, respectively.

### Cytotoxicity Bioassay

The THP-1 (acute monocytic leukemia) cells were plated at a density of 5,000 cells/well in 100 μl DMEM medium. All cell lines were incubated overnight then treated with various concentrations of purified compounds in triplicate. After cultured for 72 h, 20 μl/well of MTT solution (5 mg/ml, Sigma-Aldrich, United States) was added to each well, plate was cultured for 4 h at 37°C in a 5% CO_2_ atmosphere, which was followed by adding 150 μl DMSO to dissolve the formazan crystals, and shaking for 5 min. The absorbance was recorded at 570 nm by a microplate Reader. IC_50_ value was taken using Graph pad Prism 5 software.

## Conclusion

Common structural features of angucyclin(on)es are an angular tetracyclic benz[*a*]anthracene backbone with an aromatic D-ring and a high number of oxygen functionalities, mainly at C-1, C-7, C-8, and C-12 positions (Metsä-Ketelä et al., 2003). To the best of our knowledge, actetrophenone A (1) is the first representative of a novel-type angucyclinone bearing an nonaromatic D-ring. Actetrophenol A (2) featured a highly conjugated and aromatized tetraphene ring system, which is unprecedented for natural products. From known angucyclinones, it differs in the unusually high degree of reduction and, hence, only two oxygens in molecule. This finding provided clear proof that the oxygen atoms initially originated from molecular oxygen during biosynthesis of the benz[*a*]anthracene frame, can be removed later by the post-PKS tailoring enzymes, except for the two oxygens at C-1 and C-8 originating from acetate (Weber et al., 1994). While angucyclines are common with various sugar residues at diverse attachment positions, such as the single saccharide chains of 2, five or six sugar units attached at O-8 in landomycins (Shaaban et al., 2012), or the C-glycosidic sugar D-olivose located at C-9 in urdamycins and saquayamycins (Shaaban et al., 2011), actetrophenol B (3) is the first example of the family to contain a non-glycosylated side chain linked at the unusual C-7 position of the angucyclinone chromophore. This results in a unique *N*-acetyltryptamine-substituted tetraphene skeleton and offers new opportunities to enrich the existing structural diversity of angucyclines. Another striking difference between 3 and other angucyclines is the dynamic type of axial chirality in that atropisomers interconversion can occur spontaneously, thanks to relatively little hindrance to rotation about the unusual chiral axis as present only in actetrophenol B (3). More strikingly, actetrophenol A (2) exhibits remarkable antibiotic activity, notably including potent activity to multiple resistant *S. aureus* and *E. faecium*, consequently, actetrophenol A may be a promising new candidate to combat ESKAPE pathogens in the future.

## Data Availability

The original contributions presented in the study are included in the article/Supplementary Material, further inquiries can be directed to the corresponding author.

## References

[B1] Bringmann G. Lang G. Maksimenka K. Hamm A. Gulder T. A. M. Dieter A. (2005). Gephyromycin, the First Bridged Angucyclinone, from *Streptomyces Griseus* Strain NTK 14. Phytochemistry 66, 1366–1373. 10.1016/j.phytochem.2005.04.010 15907962

[B2] Cabrera-Afonso M. J. Lucena S. R. Juarranz Á. Urbano A. Carreño M. C. (2018). Selective Oxidative Dearomatization of Angular Tetracyclic Phenols by Controlled Irradiation under Air: Synthesis of an Angucyclinone-type Double Peroxide with Anticancer Properties. Org. Lett. 20, 6094–6098. 10.1021/acs.orglett.8b02515 30226789

[B3] Kharel M. K. Pahari P. Shepherd M. D. Tibrewal N. Nybo S. E. Shaaban K. A. (2012). Angucyclines: Biosynthesis, Mode-Of-Action, New Natural Products, and Synthesis. Nat. Prod. Rep. 29, 264–325. 10.1039/c1np00068c 22186970 PMC11412254

[B4] Liu T. Jin J. Yang X. Song J. Yu J. Geng T. (2019). Discovery of a Phenylamine-Incorporated Angucyclinone from Marine *Streptomyces* Sp. PKU-MA00218 and Generation of Derivatives with Phenylamine Analogues. Org. Lett. 21, 2813–2817. 10.1021/acs.orglett.9b00800 30924671

[B5] Ma M. Rateb M. E. Teng Q. Yang D. Rudolf J. D. Zhu X. (2015). Angucyclines and Angucyclinones from *Streptomyces* Sp. CB01913 Featuring C-Ring Cleavage and Expansion. J. Nat. Prod. 78, 2471–2480. 10.1021/acs.jnatprod.5b00601 26335269 PMC4845661

[B6] Metsä-Ketelä M. Palmu K. Kunnari T. Ylihonko K. Mäntsälä P. (2003). Engineering Anthracycline Biosynthesis toward Angucyclines. Antimicrob. Agents Chemother. 47, 1291–1296. 10.1128/aac.47.4.1291-1296.2003 12654660 PMC152523

[B8] Pescitelli G. Bruhn T. (2016). Good Computational Practice in the Assignment of Absolute Configurations by TDDFT Calculations of ECD Spectra. Chirality 28, 466–474. 10.1002/chir.22600 27098594

[B18] Rekis T. (2020). Crystallization of chiral molecular compounds: what can be learned from the Cambridge Structural Database?. Acta Cryst. 76, 307–315. 10.1107/S2052520620003601 32831251

[B9] Shaaban K. A. Ahmed T. A. Leggas M. Rohr J. (2012). Saquayamycins G-K, Cytotoxic Angucyclines from Streptomyces Sp. Including Two Analogues Bearing the Aminosugar Rednose. J. Nat. Prod. 75, 1383–1392. 10.1021/np300316b 22758660 PMC3412564

[B10] Shaaban K. A. Srinivasan S. Kumar R. Damodaran C. Rohr J. (2011). Landomycins P−W, Cytotoxic Angucyclines from Streptomyces Cyanogenus S-136. J. Nat. Prod. 74, 2–11. 10.1021/np100469y 21188999 PMC3070852

[B11] Superchi S. Scafato P. Gorecki M. Pescitelli G. (2018). Absolute Configuration Determination by Quantum Mechanical Calculation of Chiroptical Spectra: Basics and Applications to Fungal Metabolites. Cmc 25, 287–320. 10.2174/0929867324666170310112009 28294053

[B13] Weber S. Zolke C. Rohr J. Beale J. M. (1994). Investigations of the Biosynthesis and Structural Revision of Landomycin A. J. Org. Chem. 59, 4211–4214. 10.1021/jo00094a037

[B14] Wu C. van der Heul H. U. Melnik A. V. Lübben J. Dorrestein P. C. Minnaard A. J. (2019). Lugdunomycin, an Angucycline‐Derived Molecule with Unprecedented Chemical Architecture. Angew. Chem. Int. Ed. 58, 2809–2814. 10.1002/anie.201814581 PMC6519343 30656821

[B15] Xie Z. Zhou L. Guo L. Yang X. Qu G. Wu C. (2016). Grisemycin, a Bridged Angucyclinone with a Methylsulfinyl Moiety from a Marine-Derived *Streptomyces* Sp. Org. Lett. 18, 1402–1405. 10.1021/acs.orglett.6b00332 26958869

[B16] Zhang S. Yang Q. Guo L. Zhang Y. Feng L. Zhou L. (2017). Isolation, Structure Elucidation and Racemization of (+)- and (−)-pratensilins A-C: Unprecedented spiro Indolinone-Naphthofuran Alkaloids from a marine Streptomyces Sp. Chem. Commun. 53, 10066–10069. 10.1039/c7cc04983h 28840219

[B17] Zhang S. Zhang L. Fu X. Li Z. Guo L. Kou L. (2019). (+)- and (−)-actinoxocine, and Actinaphthorans A-B, C-Ring Expansion and Cleavage Angucyclinones from a marine-derived Streptomyces Sp. Org. Chem. Front. 6, 3925–3928. 10.1039/c9qo01154d

