# Supplementary material for: Ring D-Modified and Highly Reduced Angucyclinones From Marine Sediment-Derived Streptomyces sp
Source: Front Chem. 2021 Oct 12;9:756962. doi: 10.3389/fchem.2021.756962 (PMC8546756; doi:10.3389/fchem.2021.756962)
Supplement: Supplementary file 1 [file DataSheet1.docx]

Supplementary Material

Ring D-modified and highly reduced angucyclinones from marine sediment-derived *Streptomyces* sp.

Lin Guo^1†^, Qiaoli Yang^2†^, Guangfei Wang^2^, Shumin Zhang^1^, Ming Liu^1^, Xiaohong Pan^1^, Gennaro Pescitelli^3*^ and Zeping Xie^1*^

**Contents**

[Figure. S1 Structures, relative energies and populations (300K) of low-energy minima of **3** calculated at ωB97X-D/6-311+G(d,p) level with SMD solvent model for acetonitrile. 4](#_Toc79497565)

[Figure. S2 ^1^H NMR spectrum (600 MHz) of actetrophenone A (**1**) in DMSO-*d_6_*. 5](#_Toc79497566)

[Figure. S3 ^13^C NMR spectrum (150 MHz) of actetrophenone A (**1**) in DMSO-*d_6_*. 6](#_Toc79497567)

[Figure. S4 DEPT-135 spectrum (150 MHz) of actetrophenone A (**1**) in DMSO-*d_6_*. 7](#_Toc79497568)

[Figure. S5 COSY spectrum (600 MHz) of actetrophenone A (**1**) in DMSO-*d_6_*. 8](#_Toc79497569)

[Figure. S6 HSQC spectrum (600 MHz) of actetrophenone A (**1**) in DMSO-*d_6_*. 9](#_Toc79497570)

[Figure. S7 HMBC spectrum (600 MHz) of actetrophenone A (**1**) in DMSO-*d_6_*. 10](#_Toc79497571)

[Figure. S8 NOESY spectrum (600 MHz) of actetrophenone A (**1**) in DMSO-*d_6_*. 11](#_Toc79497572)

[Figure. S9 HRESIMS spectrum of actetrophenone A (**1**). 12](#_Toc79497573)

[Figure. S10 ^1^H NMR spectrum (600 MHz) of actetrophenol A (**2**) in DMSO-*d_6_*. 13](#_Toc79497574)

[Figure. S11 ^13^C NMR spectrum (150 MHz) of actetrophenol A (**2**) in DMSO-*d_6_*. 14](#_Toc79497575)

[Figure. S12 DEPT-135 spectrum (150 MHz) of actetrophenol A (**2**) in DMSO-*d_6_*. 15](#_Toc79497576)

[Figure. S13 COSY spectrum (600 MHz) of actetrophenol A (**2**) in DMSO-*d_6_*. 16](#_Toc79497577)

[Figure. S14 HSQC spectrum (600 MHz) of actetrophenol A (**2**) in DMSO-*d_6_*. 17](#_Toc79497578)

[Figure. S15 HMBC spectrum (600 MHz) of actetrophenol A (**2**) in DMSO-*d_6_*. 18](#_Toc79497579)

[Figure. S16 HRESIMS spectrum of actetrophenol A (**2**). 19](#_Toc79497580)

[Figure. S17 ^1^H NMR spectrum (600 MHz) of actetrophenol B (**3**) in CDCl_3_. 20](#_Toc79497581)

[Figure. S18 ^13^C NMR spectrum (150 MHz) of actetrophenol B (**3**) in CDCl_3_. 21](#_Toc79497582)

[Figure. S19 COSY spectrum (600 MHz) of actetrophenol B (**3**) in CDCl_3_. 22](#_Toc79497583)

[Figure. S20 HSQC spectrum (600 MHz) of actetrophenol B (**3**) in CDCl_3_. 23](#_Toc79497584)

[Figure. S21 HMBC spectrum (600 MHz) of actetrophenol B (**3**) in CDCl_3_. 24](#_Toc79497585)

[Figure. S22 NOESY spectrum (600 MHz) of actetrophenol B (**3**) in CDCl_3_. 25](#_Toc79497586)

[Figure. S23 HRESIMS spectrum of actetrophenol B (**3**). 26](#_Toc79497587)

[X-ray crystallographic analysis of actetrophenone A (**1**). 27](#_Toc79497588)

[Table S1. Crystal data and structure refinement for actetrophenone A (**1**). 27](#_Toc79497589)

[X-ray crystallographic analysis of actetrophenol B (**3**). 28](#_Toc79497590)

[Table S2. Crystal data and structure refinement for actetrophenol B (**3**). 29](#_Toc79497591)

## Figure. S1 Structures, relative energies and populations (300K) of low-energy minima of 3 calculated at ωB97X-D/6-311+G(d,p) level with SMD solvent model for acetonitrile.

|  |  |  |  |
| --- | --- | --- | --- |
| Absolute minimum (72.5%) | +0.81 kcal/mol (18.7%) | +1.62 kcal/mol (4.8%) | +1.88 kcal/mol (3.1%) |

## Figure. S2 ^1^H NMR spectrum (600 MHz) of actetrophenone A (1) in DMSO-*d_6_*.

## Figure. S3 ^13^C NMR spectrum (150 MHz) of actetrophenone A (1) in DMSO-*d_6_*.

## Figure. S4 DEPT-135 spectrum (150 MHz) of actetrophenone A (1) in DMSO-*d_6_*.

## Figure. S5 COSY spectrum (600 MHz) of actetrophenone A (1) in DMSO-*d_6_*.

## Figure. S6 HSQC spectrum (600 MHz) of actetrophenone A (1) in DMSO-*d_6_*.

## Figure. S7 HMBC spectrum (600 MHz) of actetrophenone A (1) in DMSO-*d_6_*.

## Figure. S8 NOESY spectrum (600 MHz) of actetrophenone A (1) in DMSO-*d_6_*.

## Figure. S9 HRESIMS spectrum of actetrophenone A (1).


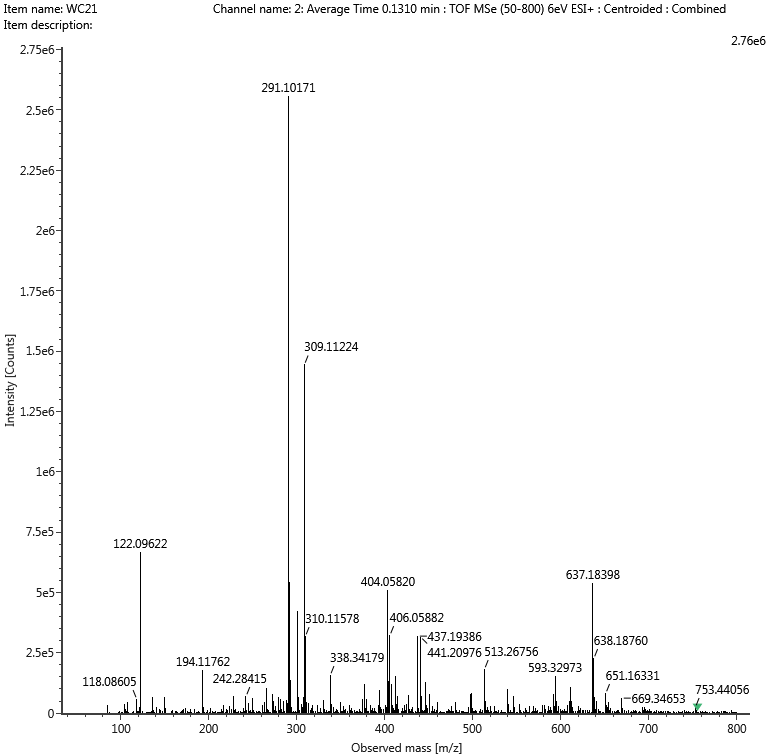


## Figure. S10 ^1^H NMR spectrum (600 MHz) of actetrophenol A (2) in DMSO-*d_6_*.

## Figure. S11 ^13^C NMR spectrum (150 MHz) of actetrophenol A (2) in DMSO-*d_6_*.

## Figure. S12 DEPT-135 spectrum (150 MHz) of actetrophenol A (2) in DMSO-*d_6_*.

## Figure. S13 COSY spectrum (600 MHz) of actetrophenol A (2) in DMSO-*d_6_*.

## Figure. S14 HSQC spectrum (600 MHz) of actetrophenol A (2) in DMSO-*d_6_*.

## Figure. S15 HMBC spectrum (600 MHz) of actetrophenol A (2) in DMSO-*d_6_*.

## Figure. S16 HRESIMS spectrum of actetrophenol A (2).


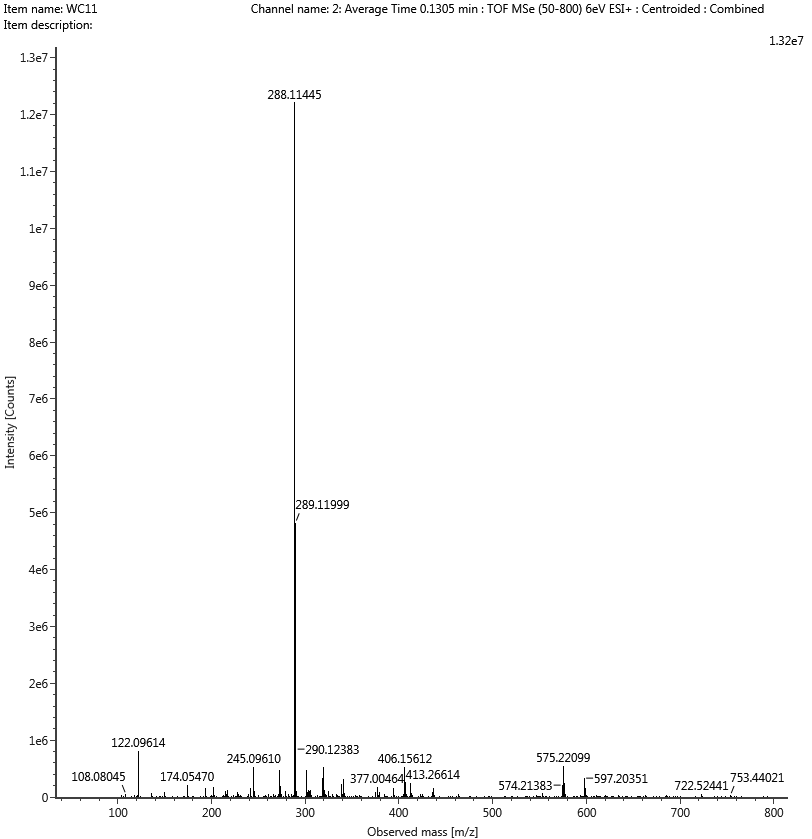


## Figure. S17 ^1^H NMR spectrum (600 MHz) of actetrophenol B (3) in CDCl_3_.

## Figure. S18 ^13^C NMR spectrum (150 MHz) of actetrophenol B (3) in CDCl_3_.

## Figure. S19 COSY spectrum (600 MHz) of actetrophenol B (3) in CDCl_3_.

## Figure. S20 HSQC spectrum (600 MHz) of actetrophenol B (3) in CDCl_3_.

## Figure. S21 HMBC spectrum (600 MHz) of actetrophenol B (3) in CDCl_3_.

## Figure. S22 NOESY spectrum (600 MHz) of actetrophenol B (3) in CDCl_3_.

## Figure. S23 HRESIMS spectrum of actetrophenol B (3).


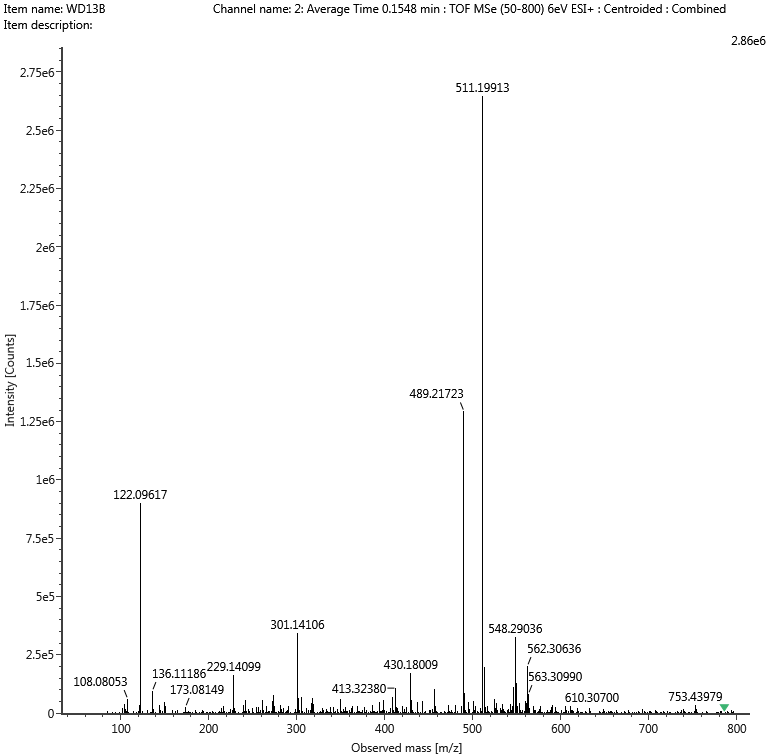


X-ray crystallographic analysis of actetrophenone A (1). Crystal data for xzp12: C_20_H_20_O_5_, *M* = 340.36, *a* = 4.9842(5) Å, *b* = 10.8892(10) Å, *c* = 14.8880(14) Å, *α* = 94.905(5)°, *β* = 92.764(5)°, *γ* = 100.032(4)°, *V* = 791.12(13) Å^3^, *T* = 100.(2) K, space group *P*-1, *Z* = 2, *μ*(Cu Kα) = 0.842 mm^-1^, 20493 reflections measured, 3145 independent reflections (*R_int_* = 0.1172). The final *R_1_*values were 0.1108 (*I*>2*σ*(*I*)). The final *wR*(*F^2^*) values were 0.2667 (*I* >2*σ*(*I*)). The final *R1* values were 0.1276 (all data). The final *wR*(*F^2^*) values were 0.2752 (all data). The goodness of fit on *F^2^* was 1.117.Crystallographic data for (±)-actetrophenone A (**1**) has been deposited in the Cambridge Crystallographic Data Centre with deposition numbers CCDC 2045687.

## Table S1. Crystal data and structure refinement for actetrophenone A (1).

| Identification code | global |
| --- | --- |
| Empirical formula | C_20_H_20_O_5_ |
| Formula weight | 340.36 |
| Temperature | 100(2) K |
| Wavelength | 1.54178 Å |
| Crystal system | Triclinic |
| space group | *P*-1 |
| Unit cell dimensions | *a* = 4.9842(5)Å*α* = 94.905(5)°.  *b* = 10.8892(10)Å*β* = 92.764(5)°.  *c* = 14.8880(14)Å*γ* = 100.032(4)°. |
| Volume | 791.12(13) Å^3^ |
| *Z* | 2 |
| Density (calculated) | 1.429 Mg/m^3^ |
| Absorption coefficient | 0.842 mm^-1^ |
| F(000) | 360 |
| Crystal size | 0.200 x 0.100 x 0.020mm^3^ |
| Theta range for data collection | 2.98 to 73.90°. |
| Index ranges | -6<=h<=5, -13<=k<=13, -18<=l<=18 |
| Reflections collected | 20493 |
| Independent reflections | 3145 [*R*(*int*) = 0.1172] |
| Completeness to theta = 73.90^o^ | 98.1 % |
| Absorption correction | Semi-empirical from equivalents |
| Max. and min. transmission | 0.98 and 0.71 |
| Refinement method | Full-matrix least-squares on *F^2^* |
| Data / restraints / parameters | 3145 / 0 / 243 |
| Goodness-of-fit on *F^2^* | 1.117 |
| Final *R* indices [*I*>2*sigma*(*I*)] | *R_1_* = 0.1108, *wR^2^* = 0.2667 |
| *R* indices (all data) | *R_1_*= 0.1276, *wR^2^* = 0.2752 |
| Largest diff. peak and hole | 0.379 and -0.451 e.Å^-3^ |

X-ray crystallographic analysis of actetrophenol B (3). Crystal data for xzp10: C_32_H_28_N_2_O_3_•4(H_2_O), *M* = 560.63, *a* = 8.9768(2) Å, *b* = 21.7920(4) Å, *c* = 14.9160(3) Å, *α* = 90°, *β* = 96.4610(10)°, *γ* = 90°, *V* = 2899.37(10) Å^3^, *T* = 100.(2) K, space group *P*121/*c*1, *Z* = 4, *μ*(Cu Kα) = 0.741 mm^-1^, 56058 reflections measured, 5726 independent reflections (*R_int_* = 0.0732). The final *R_1_* values were 0.0813 (*I*>2*σ*(*I*)). The final *wR*(*F^2^*) values were 0.2204 (*I*> 2*σ*(*I*)). The final *R_1_* values were 0.0948 (all data). The final *wR*(*F^2^*) values were 0.2340 (all data). The goodness of fit on *F^2^* was 1.041. Crystallographic data for (±)-actetrophenol B (**3**) has been deposited in the Cambridge Crystallographic Data Centre with deposition numbers CCDC 2045689.

## Table S2. Crystal data and structure refinement for actetrophenol B (3).

| Identification code | global |
| --- | --- |
| Empirical formula | C_32_H_36_N_2_O_7_ |
| Formula weight | 560.63 |
| Temperature | 100(2) K |
| Wavelength | 1.54178 Å |
| Crystal system | Monoclinic |
| space group | *P*121/*c*1 |
| Unit cell dimensions | *a* = 8.9768(2) Å*α* = 90°.  *b* = 21.7920(4) Å*β* = 96.4610(10)°.  *c* = 14.9160(3)Å*γ*=90°. |
| Volume | 2899.37(10)Å^3^ |
| *Z* | 4 |
| Density (calculated) | 1.284 Mg/m^3^ |
| Absorption coefficient | 0.741 mm^-1^ |
| F(000) | 1192 |
| Crystal size | 0.170 x 0.120 x 0.080 mm^3^ |
| Theta range for data collection | 3.61 to 72.42°. |
| Index ranges | -11<=h<=8, -26<=k<=26, -18<=l<=18 |
| Reflections collected | 56058 |
| Independent reflections | 5726 [*R*(*int*) = 0.0732] |
| Completeness to theta = 73.90^o^ | 99.9 % |
| Absorption correction | Semi-empirical from equivalents |
| Max. and min. transmission | 0.94 and 0.82 |
| Refinement method | Full-matrix least-squares on *F^2^* |
| Data / restraints / parameters | 5726 / 0 / 375 |
| Goodness-of-fit on *F^2^* | 1.041 |
| Final *R* indices [*I*>2*sigma*(*I*)] | *R_1_* = 0.0813, *wR^2^* = 0.2204 |
| *R* indices (all data) | *R_1_* = 0.0948, *wR^2^* = 0.2340 |
| Largest diff. peak and hole | 0.829 and -0.677 e.Å^-3^ |
